# Castration for Rape

**Published:** 1907-04

**Authors:** 


### Castration for Rape.

AUSTIN, TEXAS, March 20, 1907.

*Hon. C. K. Walter, House of Representatives, Austin, Texas.*

DEAR SIR: I have the honor to acknowledge the receipt of your letter of this date, in which you propound the following interrogatories relative to House bill No. 191, providing for castration as an additional penalty for rape; and request my opinion:

1. "Do you think if the Penal Code is amended so as to provide for castration, that it will have a tendency to reform the offender and to deter others from committing said offenses?

2. "In your opinion can castration be performed in such a manner as to deprive the offender of all erectile power?

3. "Do you consider this additional punishment 'cruel and unusual?'"

Replying, seriatim, I beg to say:

1. It is amply demonstrated that capital punishment, swift and sure, even lynching by rope, bullet or the fagot, has failed to prevent or diminish the crime of rape. On the contrary, it is on the increase. In my opinion, if any measure would have a deterrent effect on those disposed to the crime, it would be castration plus a penitentiary sentence.

Castration for this offense should not be regarded as a punishment, so much as a remedial agency, both curative (for I regard the propensity to rape children, especially of a different race, as a perversion of the fundamental instinct of procreation) and preventive, and you may say also, punitive. It would certainly "reform the offender," inasmuch as it would deprive him of the power to repeat it.

2. It can be done so as to deprive him of all erectile power.

3. I do not consider castration for rape by any means a cruel and unusual punishment. There would be no torture, no physical suffering. It should be done by a skillful surgeon, and would be done under complete anesthesia. Unusual, it may be; but, as said before, it is not to be regarded so much as a punishment as a sanitary police measure in the interest of public morals and public safety.

In the Kansas Asylum for Feeble Minded, at Winfield, castration for other forms of sexual perversion has been practiced with success, with the sanction of the law. The late Governor Hogg gave it as his opinion that the surgeon of any institution has the right to do this operation, the same as he would any other operation, for relief or cure of any disease. I have the honor to be

Yours very truly,

F. E. Daniel, M. D.

House bill No. 191 is as follows (by Walter of Gonzales):

*An Act to amend Article 639 of Chapter 7, Title XV of the Penal Code of the State of Texas, in reference to the punishment for rape. Providing how the operation is to be performed, by whom and how paid for.*

*Be it enacted by the Legislature of the State of Texas:*

Section 1. That Article 639 of Chapter 7, Title XV of the Penal Code of the State of Texas, be and the same is hereby amended so as to read as follows:

Article 639. Whoever shall be guilty of rape by force shall be punished by death or by confinement in the penitentiary for life, or for any term of years, not less than two, or by castration, or by such imprisonment and castration, in the discretion of the jury.

The bill was amended so as to make it the duty of the sheriff, by order of the court, to have the county physician of the county where the crime occurred to perform the operation, and he is to be paid $25 by the State.

As amended the bill passed the House by practically a unanimous vote, there being but two dissenting voices. It was sent to the Senate where, should there be time before adjournment (April 12), it is thought it will receive little or no opposition. At this writing it is greatly feared the bill will not be reached. (P. S.: Died on the calendar; adjourned April 12th.)

Judge Walter made a ringing speech in supporting the bill. He impressed upon his hearers that this is the question of the hour! The crime is on the increase despite most drastic measures, and if we would avert a race war of extermination *something* should be done. Let every husband, father, brother, lover, bring it home to himself! When will this blight strike your home? This measure should have the support of every man who has wife, mother, sister, daughter or sweetheart! And it did! All honor to the able and courageous champion of the protection of our homes, the Hon. C. K. Walter of Gonzales!

